# Minimally Invasive Transforaminal Lumbar Interbody Fusion at L5-S1 through a Unilateral Approach: Technical Feasibility and Outcomes

**DOI:** 10.1155/2016/2518394

**Published:** 2016-06-28

**Authors:** Won-Suh Choi, Jin-Sung Kim, Kyeong-Sik Ryu, Jung-Woo Hur, Ji-Hoon Seong

**Affiliations:** Department of Neurosurgery, Seoul St. Mary's Hospital, Catholic University, Seoul 06591, Republic of Korea

## Abstract

*Background*. Minimally invasive spinal transforaminal lumbar interbody fusion (MIS-TLIF) at L5-S1 is technically more demanding than it is at other levels because of the anatomical and biomechanical traits.* Objective*. To determine the clinical and radiological outcomes of MIS-TLIF for treatment of single-level spinal stenosis low-grade isthmic or degenerative spondylolisthesis at L5-S1.* Methods*. Radiological data and electronic medical records of patients who underwent MIS-TLIF between May 2012 and December 2014 were reviewed. Fusion rate, cage position, disc height (DH), disc angle (DA), disc slope angle, segmental lordotic angle (SLA), lumbar lordotic angle (LLA), and pelvic parameters were assessed. For functional assessment, the visual analogue scale (VAS), Oswestry disability index (ODI), and patient satisfaction rate (PSR) were utilized.* Results*. A total of 21 levels in 21 patients were studied. DH, DA, SLA, and LLA had increased from their preoperative measures at the final follow-up. Fusion rate was 86.7% (18/21) at 12 months' follow-up. The most common cage position was anteromedial (15/21). The mean VAS scores for back and leg pain mean ODI scores improved significantly at the final follow-up. PSR was 88%. Cage subsidence was observed in 33.3% (7/21).* Conclusions*. The clinical and radiologic outcomes after MIS-TLIF at L5-S1 in patients with spinal stenosis or spondylolisthesis are generally favorable.

## 1. Introduction

Transforaminal lumbar interbody fusion (TLIF) has been a commonly used surgical option for treating various kinds of degenerative lumbar spinal pathologies that require fusion [1–3]. In recent years, TLIF using minimally invasive techniques has gained popularity with the advancement of minimally invasive spinal techniques and instruments, such as tubular retractors and percutaneous pedicle screw fixation (PPF) [4–8]. Other minimally invasive interbody fusion techniques such as anterior lumbar interbody fusion and axial lumbar interbody fusion [9, 10] at the L5-S1 level are also utilized to treat degenerative lumbar spines. Still, MIS-TLIF remains one of the more popular surgical procedures because of surgeons' anatomic familiarity with the posterior approach, the reduced neural tissue retraction compared with PLIF, and the reduced trauma to back muscles and bony structures compared with open TLIF or PLIF [8, 11–13].

The most common levels for MIS-TLIF surgery are L4-5 and L5-S1 because of the high rates of clinically significant spinal stenosis, or degenerative or isthmic spondylolisthesis at these levels [5, 14]. Despite the benefits of MIS-TLIF, some surgeons are doubtful that this method should be employed for fusion at the L5-S1 level because of its unique anatomical and biomechanical attributes. Although numerous studies have reported on comparisons between L5-S1 and other lumbar spine levels in terms of functional and clinical outcomes for PLIF and other conventional methods [15–17], to our knowledge no studies have yet reported on the clinical and radiological outcomes of instrumented MIS-TLIF focused on L5-S1 for treating various types of unstable lumbar spondylosis. This study aims to evaluate the radiological as well as functional outcomes of MIS-TLIF at the L5-S1 level.

## 2. Material and Methods

### 2.1. Study Design

From May 2012 to August 2014, 22 consecutive patients who had been diagnosed with degenerative spinal diseases at the L5-S1 segment underwent MIS-TLIF at our institution. The institutional review board at our hospital approved of this study and standardized work-up protocol. The inclusion criteria were as follows: (1) the presence of unstable, single-level, low-grade (Meyerding grade I or II), isthmic spondylolisthesis, or (2) degenerative spondylosis including degenerative spondylolisthesis, foraminal stenosis with central stenosis, degenerative disc disease, and recurrent disc herniation, (3) persistence of symptoms that correlated with the radiological findings despite conservative treatment for at least 6 weeks, (4) index surgical level of L5-S1 only, and (5) minimum follow-up period of at least 12 months. Patients with metabolic bone disease, infection, spinal trauma, tumors, and multilevel fusion were excluded from the study.

Functional and clinical preoperative data were obtained from patients' medical records. Body mass index (BMI) of the patients was calculated from medical records and bone density was assessed with dual energy X-ray absorptiometry. Preoperative radiological evaluation consisted of standing anteroposterior and lateral radiographs, standing whole spine radiograph, magnetic resonance imaging (MRI), and computed tomography (CT) of the lumbar spine in all patients. Postoperative radiographs were taken at immediate operative period, as well as 6 and 12-month follow-up visits. CT scan of the lumbar spine was performed at 6 and 12 months' follow-up and annually thereafter in all patients.

### 2.2. Radiological Assessment

**Figure 1:**
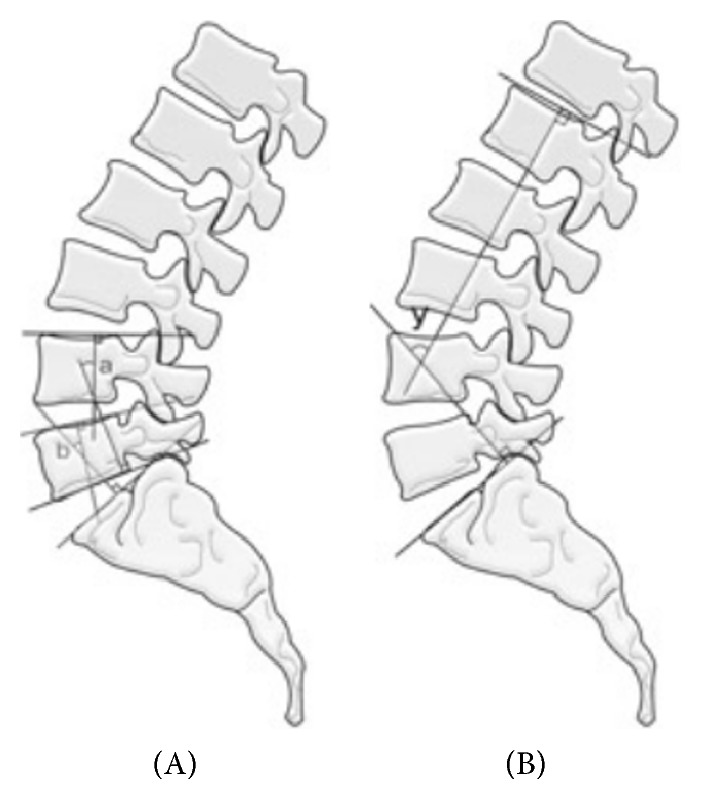
a: segmental lordotic angle (SLA), b: SLA of level L5-S1, and y: lumbar lordotic angle (LLA).

**Figure 2:**
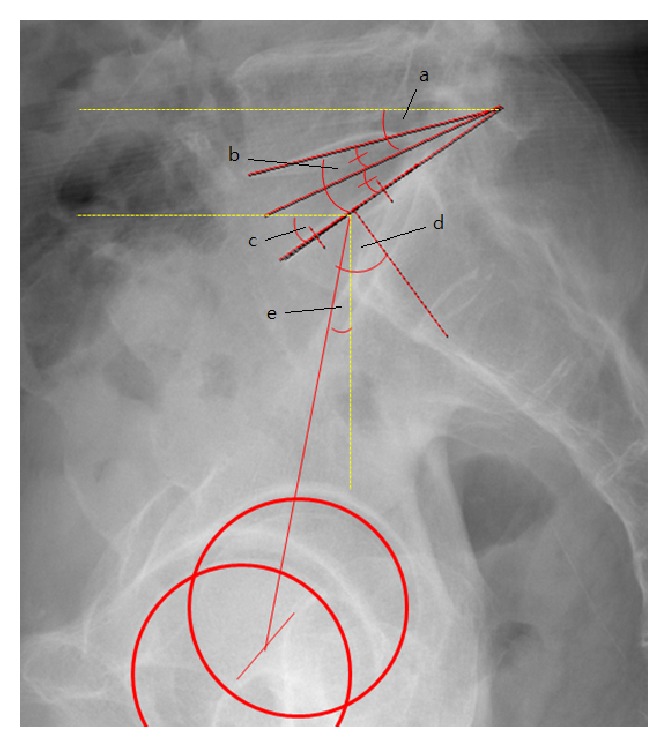
a: disc slope angle (DSA), b: disc angle (DA), c: sacral slope (SS), d: pelvic incidence (PI), and e: pelvic tilt (PT).

Disc angle (DA), segmental lordotic angle (SLA), lumbar lordotic angle (LLA), disc slope angle (DSA), and pelvic parameters were also measured in preoperative standing whole spine radiographs. The DA was measured as the angle subtended by lines parallel to the lower endplate of L5 and the upper endplate of S1. DSA was measured as the angle between a horizontal line and a line connecting the midpoints of the anterior and posterior disc spaces on standing whole spine lateral radiographs [15]. LL was measured between the superior endplate of L1 and the upper endplate of the sacrum (Figure 1). As for pelvic parameters, the PI was measured as the angle between the line perpendicular to the sacral plate at its midpoint and the line connecting this point to the axis of the femoral heads. The SS was measured as the angle between the superior plate of S1 and a horizontal line. The PT was measured as the angle between the line connecting the midpoint of the sacral plate to the femoral heads axis and the vertical line (Figure 2) [18, 19]. DA, SLA, LLA, DSA, and pelvic parameters were measured preoperatively, immediately postoperatively, and at each subsequent outpatient follow-up visit at 6 and 12 months in all patients and in additional follow-up images when they were available.

**Figure 3:**
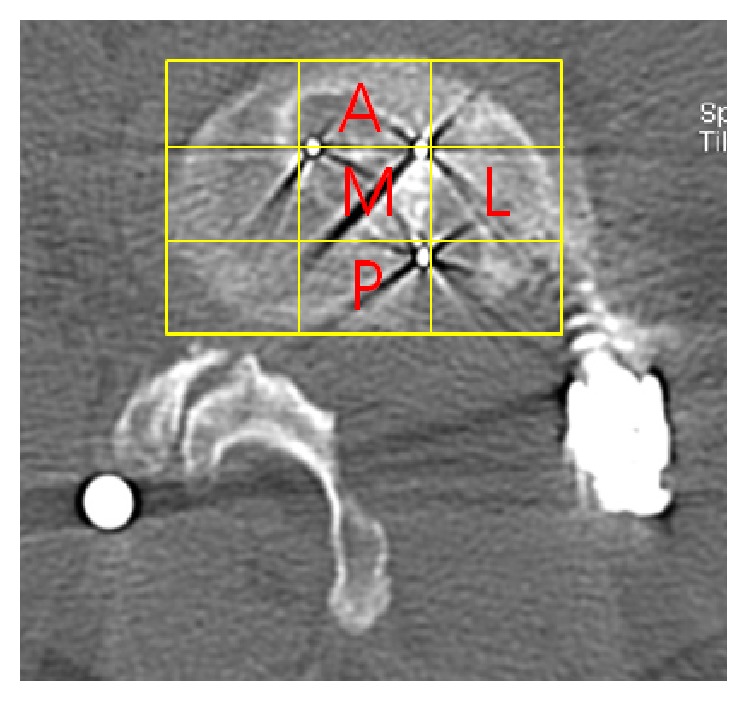
A: anterior, M: medial, L: lateral, and P: posterior.

Modified Bridwell fusion criteria [20, 21] (Table 1) for the lumbar spine were used to assess fusion on CT scan of the lumbar spine at 6 and 12 months after operation, and additional annual follow-up CT scans were also analyzed when available. Only grades I and II were considered satisfactory fusion. Position of the cage on the axial CT scan image was analyzed using a 3 × 3 grid system. The axial image of the vertebral body was divided into 9 segments by overlapping a 3 × 3 grid onto the image, and the area of the grid occupied by the interbody cage was assessed (Figure 3). Any cage subsidence or endplate violation, defined as 2 mm or more migration of the interbody cage into the adjacent vertebral bodies on coronal and sagittal CT reconstruction images, was also noted when identified during postoperative follow-up examinations.

All measurements were independently done by two spine fellows using images stored on a picture archiving and communication system (Maroview, Marosis Co., Seoul, Korea).

### 2.3. Clinical Assessment

Clinical and functional outcomes were measured using the visual analogue scale (VAS), the Oswestry disability index (ODI), and the patient satisfaction rate (PSR). All clinical and functional assessments were conducted by one clinical research assistant.

### 2.4. Surgical Technique

**Figure 4:**
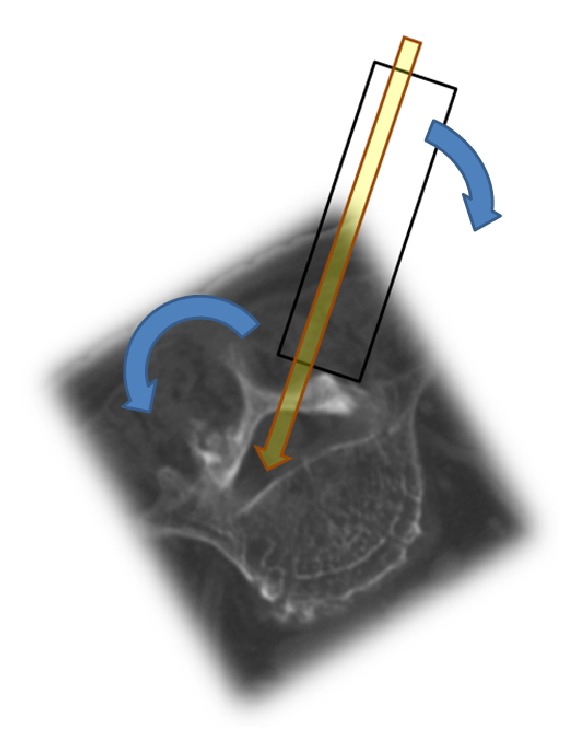
By angulating the tubular retractor so that its distal end is facing more towards the opposite side of the surgeon, visualization and access to the contralateral side are improved. Tilting the table towards the opposite side, as seen in the figure, can help the surgeon maintain a more natural posture, as the retractor would be more perpendicular to the floor.

All operations were performed by one senior author (JS Kim). All MIS-TLIF procedures were done via unilateral approach. Under fluoroscopic guidance, a 2~3 cm paraspinal skin incision is made between the L5 and S1 pedicles on anteroposterior image. After an incision is made on the lumbodorsal fascia between the multifidus and longissimus muscles, sequential widening of the incision is made using tubular dilators (Insight Access Retractor System, DePuy-Synthes Spine, Massachusetts, USA), and a 24 mm working channel is docked. Under microscope visualization, total facetectomy and partial laminectomy are performed using a combination of osteotome and high-speed burr and Kerrison rongeurs. The ligamentum flavum is resected and nerve root is retracted medially. Complete discectomy is performed, and meticulous preparation of the central and contralateral endplates is performed with angled ring curettes. Patients with bilateral foraminal stenosis on MRI or CT with corresponding symptoms underwent bilateral decompression through the unilateral laminofacetectomy site. This is done by resecting portions of the contralateral inferior articular process, lamina, and ligamentum flavum through the corridor created by ipsilateral laminofacetectomy. In order to facilitate better visualization of the contralateral side, the tubular retractor needs to be angled so that the distal end of the retractor is facing the base of the spinous process, away from the surgeon. Tilting the table away from the surgeon after repositioning the retractor can help the surgeon maintain a more natural and comfortable posture during the operation (Figure 4).

After completion of discectomy and foraminal decompression, a banana-shaped cage (Crescent, Medtronic Sofamor Danek, Tennessee, USA) or straight cage (Opal, DePuy-Synthes Spine, Massachusetts, USA) filled with morselized bone fragments obtained from laminofacetectomy mixed with demineralized bone matrix (DBM) is inserted into the disk space. The disk space ventral to the inserted cage is also packed with morselized bone and DBM. Then, percutaneous pedicle screws (Sextant, Medtronic Sofamor Danek, Tennessee, USA, or Viper 2, DePuy-Synthes Spine, Massachusetts, USA) are inserted under fluoroscopic guidance, and adequate sized rods are fitted. The wounds are sufficiently irrigated, drainage catheters are placed on the side of approach in every case, and the wounds are sutured layer by layer. The type of cage and percutaneous screw system to be used are selected via randomization as part of another ongoing prospective study.

### 2.5. Statistical Analysis

The baseline data and preoperative DH, DA, SLA, LLA, DSA, and pelvic parameters for fusion and nonunion were analyzed using either the paired *t*-test or Wilcoxon signed-rank test.

A *p* < 0.05 was considered significant. All statistical analysis was performed using SPSS version 21.0 (IBM Corporation, Armonk, NY, USA).

## 3. Results

A total of 22 patients (7 male, 14 female) met the inclusion criteria. One patient was lost to follow-up (drop rate 4.5%). There were a total of 21 operated levels. The mean age of patients was 61.5 ± 11.2 years. Mean follow-up period was 17.9 ± 8.8 (12–36) months. On MRI of the lumbar spine, 12 patients had spinal stenosis without spondylolisthesis, 8 patients had spinal stenosis with spondylolisthesis, and 2 patients had foraminal stenosis. Of the patients with spondylolisthesis, 6 were of degenerative type, and 2 were of isthmic types. Sixteen patients were operated on via left-side approach, and five via right-side approach. One of the patients with foraminal stenosis had previously undergone microdiscectomy for extraforaminal herniated disc, but the herniation had recurred. The cages used were banana-shaped in 38.1% (8/21) of cases and straight in the remaining 61.9% (13/21). Bilateral decompression through unilateral facetectomy site was performed in 23.8% (5/21) of the patients. The demographic data on the patients are summarized in Table 2. Mean operating time was 126.4 ± 30.9 minutes from skin incision to final wound closure, and mean blood loss was 212 ± 90.1 cc. Mean hospital stay postoperatively was 7.1 ± 3.3 days. There was 1 case of dural tear during facetectomy and 1 case of screw malposition (Table 2).

Radiographic evidence of fusion according to the modified Bridwell criteria (grade I and grade II) was observed in 95.2% (20/21) of the patients in the 12-month follow-up CT of the lumbar spine. Cage subsidence was observed in 33.3% (7/21) of patients (Table 3). Mean DSA measured from preoperative standing radiograph of the lumbar spine was 30.45. DH, DA, SLA, and LLA significantly increased from preoperative measures at the final follow-up measurements. PI and PT showed significant postoperative decrease, and SS showed significant increase (Table 3).

On axial CT images taken postoperatively, cage position was anteromedial in 61.9% (13/21), mediolateral in 23.8% (5/21), medial in 9.5% (2/21), and posteromedial in 4.8% (1/21) of the patients.

**Figure 5:**
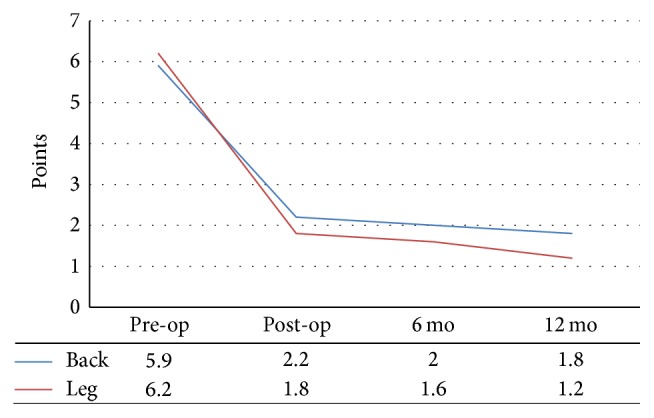
VAS for back and leg pain.

**Figure 6:**
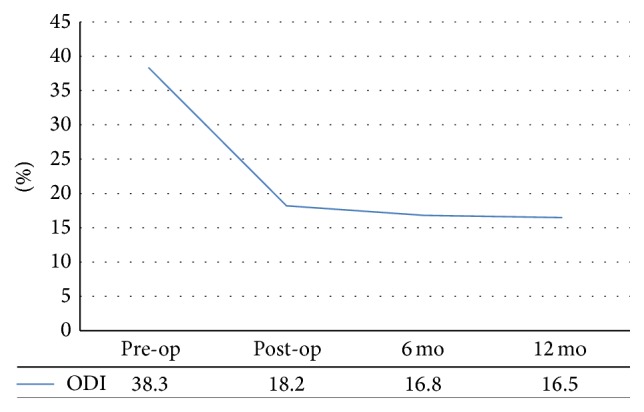
ODI scores.

Patients were ambulated 6~8 hours postoperatively to assess their postoperative day functional outcomes. The mean VAS scores for back and leg pain decreased from 5.9 and 6.2 at baseline to 1.8 and 1.2 at the final follow-up, respectively (Figure 5), and mean ODI scores improved from 38.3% to 16.5% at final follow-up (Figure 6). PSR at final follow-up was 88%, 19.0% (4/21) had additional nerve blocks, and 1 patient underwent reoperation to reposition a malpositioned screw.

## 4. Discussion

Minimally invasive approaches are the new trend in spinal fusion surgery. MIS-TLIF has gained popularity over the years with the advantages of smaller incisions, reduced trauma to paraspinal muscles, decreased intraoperative blood loss, shorter hospital stays, and decreased rates of operative site infection, all of which contribute to lower postoperative morbidity and expedited postoperative recovery [22–28]. Multiple studies have reported favorable results after treatment of DS with MIS-TLIF [13, 29, 30]. L5-S1 is one of the most affected levels of the spine in spondylolisthesis, especially in patients with IS. Treatment of clinically significant IS using the TLIF method has yielded favorable results according to some studies [31, 32]. However, MIS-TLIF via unilateral approach with or without bilateral decompression at level L5-S1 may present a challenge to the surgeon because of its unique anatomical and biomechanical properties.

**Figure 7:**
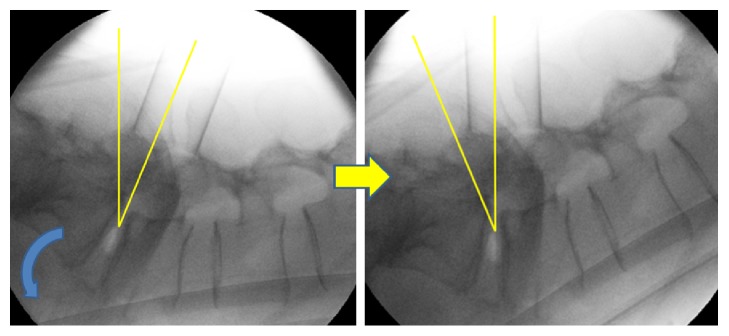
Because of the orientation of the L5-S1 disc space, the tubular retractor when placed in line with the disc space can often be slanted. Resultant unnatural posture of the surgeon can cause fatigue to the surgeon. To avoid this, we tilt the operating table caudally so the tubular retractor is almost perpendicular to the floor.

**Figure 8:**
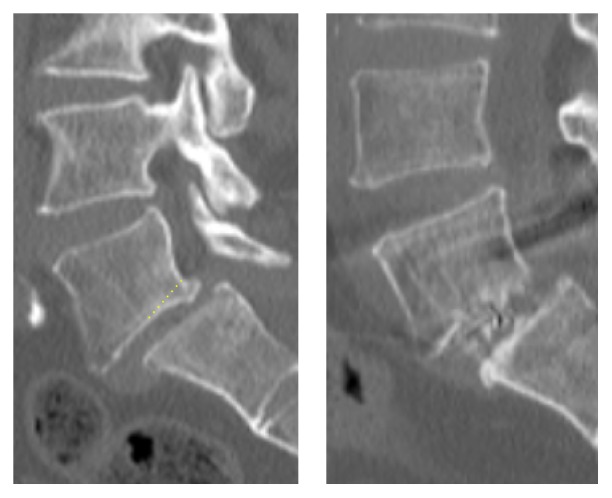
Posterior tip of caudal endplate of L5 was drilled slightly to widen the opening for cage insertion.

First, the DSA of L5-S1 is the greatest among all levels of the lumbar spine. In our single cohort group, the mean DSA was 30.5°, which is more than twice the mean L4-5 DSA that has been reported in previous published literature [15]. With the patient in the prone position on operating table, high DSA means that, in order obtain the ideal trajectory into the L5-S1 disk space, the tubular retractor has to be tilted more cranially than for other levels of the lumbar spine, and the surgeon's resulting posture could be uncomfortable, especially for inexperienced surgeons (Figure 7). Second, the L5-S1 disk space is conical in shape, with the posterior margin being narrower than anterior margin, compared with other lumbar levels [15, 33]. In our cohort, the preoperative DA was 8.8° and DH was 7.0 mm. In order to obtain maximal and tight contact between the interbody cage and the bony endplates, a tall cage with high lordotic angle should be used, but such tall cages are difficult to insert through the narrow posterior disc space. In order to overcome this difficulty, we drilled out the posterior edges of the caudal endplates of L5 and sometimes the posterior edges of the cranial endplates of S1, thereby widening the posterior disc space (Figure 8). There are reports that the cranial endplate is structurally weaker than the caudal endplate [34], so the cranial endplate of S1 was not drilled unless the posterior disc space was excessively narrow. Sometimes, the upper margins of S1 pedicles also had to be drilled. Because S1 pedicles usually have larger diameters than other levels, we believe that our drilling 2–4 mm of the upper part of S1 pedicles did not harm their integrity, and follow-up images did not show any evidence of S1 screw loosening in any of the patients. Extra care should still be taken to avoid unnecessary drilling of the endplates, as it could directly result in endplate violation during cage insertion or subsidence and migration afterwards. Third, the interpedicular distance between L5 and S1 is wide compared with other lumbar levels. When performing contralateral foraminal decompression through a unilateral facetolaminectomy corridor, the distance to the contralateral foramen is longer than it is in other lumbar levels. We tilted the tubular retractor laterally toward the surgeon and also tilted the operating table to the contralateral side to obtain a good working channel view. Then, gentle drilling of the inferior articular process and laminar was done using a high-speed diamond burr. To achieve adequate discectomy and endplate preparation of the contralateral side, we used angled curettes and angled box curettes that have longer reach to the contralateral side and are well suited for MIS-TLIF. Lastly, the dorsal root ganglion (DRG) has the largest diameter at L5, and the center of the DRG is also located at the lateral zone of the foramen at L5 [35]. This means that the entry site for cage insertion is often obscured by L5 DRG or L5 root, especially if it is enlarged or edematous. In order to avoid excessive retraction of nerve roots and resultant postoperative neurological deficit, the lower border of the L5 transverse process and upper border of S1 were gently drilled, widening the approach corridor to the disc space.

A number of studies have reported lower fusion rates at L5-S1 than at other levels. In a study by Agazzi et al., the fusion rate at L4-5 was 96.2% and at L5-S1, it was 95.2% (20 of 21) [16]. Ito et al. also reported a fusion rate of 96.4% (80 of 83) at L4-5 and 87.5% (7 of 8) at L5-S1 [36]. In many of the documented studies, the fusion rate at L5-S1 tended to be lower, which could have been because of the high DSA at L5-S1 and the resultant high shear force, high range of motion, and high DH and DA. In order to maximize the contact area and contact force between the interbody cage and the bony endplates, we used long cages, usually 32 mm in length, and pushed the cage as anteriorly as possible. The location of the cage on the CT scan shows that cage position was anteromedial as intended in the majority of the cases. We also packed bone chips mixed with DBM ventral to the inserted interbody cage to facilitate fusion. The fusion rate on 1-year follow-up CT was 95.2% according to the modified Bridwell fusion criteria for posterior fusion surgery, which is comparable with or higher than previously published results.

Our study has a number of limitations. First, it is a retrospective study, and the number of enrolled patients was small. Second, all operations were done by a single experienced surgeon, and the results might have been different if they had been performed by a less experienced surgeon. Third, although fusion rates and subsidence rates were compared to those of L4-5 level, no comparison of other sagittal and spinopelvic parameters of L5-S1 level with other levels was made. Similarly, no comparison between MIS-TLIF and other conventional fusion methods such as PLIF at L5-S1 level there was made, although we aim to perform a randomized clinical trial comparing the results of PLIF at L5-S1 with MIS-TLIF at L5-S1 in the near future.

## 5. Conclusion

Despite technical difficulties arising from the anatomical and biomechanical traits of L5-S1, our results for MIS-TLIF at this level show satisfactory fusion rates and functional outcomes. Changes in DH and SLA were also significant.

## Figures and Tables

**Table 1:** 

Grade I	Fused with remodeling and trabeculae present

Grade II	Graft intact, not fully remodeled and incorporated, but no lucency present

Grade III	Graft intact, potential lucency present at top and bottom of graft

Grade IV	Fusion absent with collapse/resorption of the graft

**Table 2:** Demographic characteristics of the patients.

Characteristics	
*Age*	61.5 ± 11.2 (29–71)
*Sex*	Male: 7 (33.3%); female: 14 (66.7%)
*BMI* (kg/m^2^)	26.5 ± 7.1 (17.0–45.9)
*Bone density* (*T*-score)	−1.6 ± 0.8 (−4.0–1.5)
*Primary diagnosis*	
Spinal stenosis without spondylolisthesis	12 (57.1%)
Isthmic spondylolisthesis	2 (9.5%)
Degenerative spondylolisthesis	6 (28.6%)
Foraminal stenosis	3 (14.3%)
Adjacent segment pathology	1 (4.8%)
Recurrent disc herniation	1 (4.8%)
*Comorbid conditions*	Diabetes mellitus: 11 (52.3%)
	Hypertension: 10 (47.6%)
*Cage type*	Banana-shaped: 8 (38.1%); straight: 13 (61.9%)
*Bilateral decompression through unilateral facetectomy*	5 (23.8%)
Perioperative parameter	
*Operation time* (mins)	126.4 ± 30.9 (110–155)
*Blood loss* (mL)	212 ± 90.1 (200–450)
*Postoperative hospital stay* (days)	7.1 ± 3.3 (6–12)
*Complications*	2 (14.3%)
	(1: dural tear; 1: screw malposition)

**Table 3:** Lumbopelvic parameters.

	Preoperative	Immediate postoperative	6 months	12 months
DH	7.0 ± 1.9 (3.6–11.1)	8.8 ± 1.8 (5.8–12.8)	8.2 ± 1.4 (5.7–10.9)	7.8 ± 1.3 (6.0–10.2)
SLA	13.3 ± 4.3 (6.1–21.1)	16.5 ± 4.9 (9.1–23.4)	15.6 ± 5.9 (8.5–23.1)	15.5 ± 4.4 (7.5–23.2)
LLA	44.3 ± 12.3 (23.9–71.9)	48.6 ± 12.8 (24.6–82)	47.6 ± 13.9 (25.6–77)	47.1 ± 13.2 (26.9–76.3)
DA	8.8 ± 3.5 (1.7–14.2)	10.8 ± 3.05 (4.8–15)	—	—
DSA	30.5 ± 7.7 (20.6–46.4)	—	—	—
PI	54.7 ± 9.7 (33.5–57.5)	54.3 ± 8.8 (32.2–61.6)	—	—
PT	18.9 ± 9.9 (9.1–39.4)	17.4 ± 8.8 (6–29.1)	—	—
SS	34.1 ± 6.9 (22.6–44.3)	35.2 ± 8.1 (18.9–49.2)		
Fusion rate	—	—	67.7	95.2
Endplate violation/cage subsidence (cumulative)	—	5	6	7
